# A Simple Genetic Architecture Underlies Morphological Variation in Dogs

**DOI:** 10.1371/journal.pbio.1000451

**Published:** 2010-08-10

**Authors:** Adam R. Boyko, Pascale Quignon, Lin Li, Jeffrey J. Schoenebeck, Jeremiah D. Degenhardt, Kirk E. Lohmueller, Keyan Zhao, Abra Brisbin, Heidi G. Parker, Bridgett M. vonHoldt, Michele Cargill, Adam Auton, Andy Reynolds, Abdel G. Elkahloun, Marta Castelhano, Dana S. Mosher, Nathan B. Sutter, Gary S. Johnson, John Novembre, Melissa J. Hubisz, Adam Siepel, Robert K. Wayne, Carlos D. Bustamante, Elaine A. Ostrander

**Affiliations:** 1 Department of Genetics, Stanford School of Medicine, Stanford, California, United States of America; 2 Department of Biological Statistics and Computational Biology, Cornell University, Ithaca, New York, United States of America; 3 Cancer Genetic Branch, National Human Genome Research Institute, National Institutes of Health, Bethesda, Maryland, United States of America; 4 Department of Ecology and Environmental Biology, University of California, Los Angeles, California, United States of America; 5 Affymetrix Corporation, Santa Clara, California, United States of America; 6 Department of Clinical Sciences, College of Veterinary Medicine, Cornell University, Ithaca, New York, United States of America; 7 Department of Veterinary Pathobiology, University of Missouri, Columbia, Missouri, United States of America; Harvard University, United States of America

## Abstract

Domestic dogs exhibit tremendous phenotypic diversity, including a greater variation in body size than any other terrestrial mammal. Here, we generate a high density map of canine genetic variation by genotyping 915 dogs from 80 domestic dog breeds, 83 wild canids, and 10 outbred African shelter dogs across 60,968 single-nucleotide polymorphisms (SNPs). Coupling this genomic resource with external measurements from breed standards and individuals as well as skeletal measurements from museum specimens, we identify 51 regions of the dog genome associated with phenotypic variation among breeds in 57 traits. The complex traits include average breed body size and external body dimensions and cranial, dental, and long bone shape and size with and without allometric scaling. In contrast to the results from association mapping of quantitative traits in humans and domesticated plants, we find that across dog breeds, a small number of quantitative trait loci (≤3) explain the majority of phenotypic variation for most of the traits we studied. In addition, many genomic regions show signatures of recent selection, with most of the highly differentiated regions being associated with breed-defining traits such as body size, coat characteristics, and ear floppiness. Our results demonstrate the efficacy of mapping multiple traits in the domestic dog using a database of genotyped individuals and highlight the important role human-directed selection has played in altering the genetic architecture of key traits in this important species.

## Abstract 2

The largest genetic study to date of morphology in domestic dogs identifies genes controlling nearly 100 morphological traits and identifies important trends in phenotypic variation within this species.

## Author Summary

Dogs offer a unique system for the study of genes controlling morphology. DNA from 915 dogs from 80 domestic breeds, as well as a set of feral dogs, was tested at over 60,000 points of variation and the dataset analyzed using novel methods to find loci regulating body size, head shape, leg length, ear position, and a host of other traits. Because each dog breed has undergone strong selection by breeders to have a particular appearance, there is a strong footprint of selection in regions of the genome that are important for controlling traits that define each breed. These analyses identified new regions of the genome, or loci, that are important in controlling body size and shape. Our results, which feature the largest number of domestic dogs studied at such a high level of genetic detail, demonstrate the power of the dog as a model for finding genes that control the body plan of mammals. Further, we show that the remarkable diversity of form in the dog, in contrast to some other species studied to date, appears to have a simple genetic basis dominated by genes of major effect.

## Introduction

The vast phenotypic diversity of the domestic dog, its unique breed structure, and growing genomic resources present a powerful system for genetic dissection of traits with complex inheritance (reviewed in [1]). In the past three years, dozens of genetic variants and Quantitative Trait Loci (QTL) have been identified which influence breed-defining traits including those for skeletal size [2], coat color [3],[4], leg length [5], hairlessness [6], wrinkled skin [7], hair length, curl, and texture [8], and presence of a dorsal fur ridge [9]. Here, we describe the development and application of a high-density map of common genetic variation in the domestic dog (the “CanMap Project”). We simultaneously delineate genomic regions underlying 57 morphological traits defined at the breed level, including body weight, absolute and relative length and width of long bones, absolute and proportional skull length and width, teeth characters, and a key domestication correlate—prick versus floppy ears (see Figure S1).

We are particularly interested in assessing whether the majority of phenotypic variation among breed-affiliated dogs is a consequence of QTLs of large effect or whether much of the variation is attributable to many QTLs of modest or small effect. The latter situation resembles the emerging picture from genome-wide association studies in humans, laboratory animals, and outcrossed domesticated plants such as maize [10],[11]. In those systems, the genetic architecture of most phenotypes tested to date—including body size, body mass index (BMI), lipid level, and flowering time—appear to be under the control of hundreds of genes, each contributing a very modest amount to the overall heritability of the trait. The alternative model is that mutations of large phenotypic effect underlie most of these traits in dogs and that the same variants have been transferred to a wide diversity of dog breeds leading to phenotypic diversity from a narrow genetic base [5],[8],[12].

To distinguish between these two genetic models and to understand the extent to which domestication and artificial selection have shaped the dog genome, we genotyped more than 120,000 potential single nucleotide polymorphisms using DNA isolated from 915 dogs representing 80 American Kennel Club (AKC) recognized breeds as well as 83 wild canids that included wolves, jackals, and coyotes and 10 Egyptian shelter dogs [13]. We developed a new genotype-calling algorithm for Affymetrix array data (MAGIC) that relaxes key assumptions and limitations of current callers such as Hardy-Weinberg equilibrium among genotype clusters. This dramatically improved the performance of the Affymetrix v2 Canine GeneChip, producing 99.9% concordance across 154 technical replicates for 60,968 SNPs (see Methods). The high density of markers and the inclusion of wild canids and outbred village dogs allowed for unprecedented resolution of the effect of domestication and artificial selection on the dog genome. Detailed results can be obtained from the Canine SNP browser track hosted at http://genome-mirror.bscb.cornell.edu/.

## Results

### Genomic Signatures of Dog Demography History

To investigate how human-directed breeding has altered the landscape of the dog genome, we quantified pairwise SNP linkage disequilibrium (LD), haplotype diversity across 15-SNP windows (as inferred by fastPhase [14]) and runs of homozygosity (ROHs) greater than 1 Mb for each individual (indicative of autozygosity) using the genotype data from the 59 breeds with ≥10 individuals and a population of village dogs and wolves (see Methods). Long ROHs are a product of recent inbreeding, indicative of contemporary population size and mating system, whereas haplotype diversity and LD across shorter genomic scales (<1 Mb) are informative of more ancient population processes.

**Figure 1:**
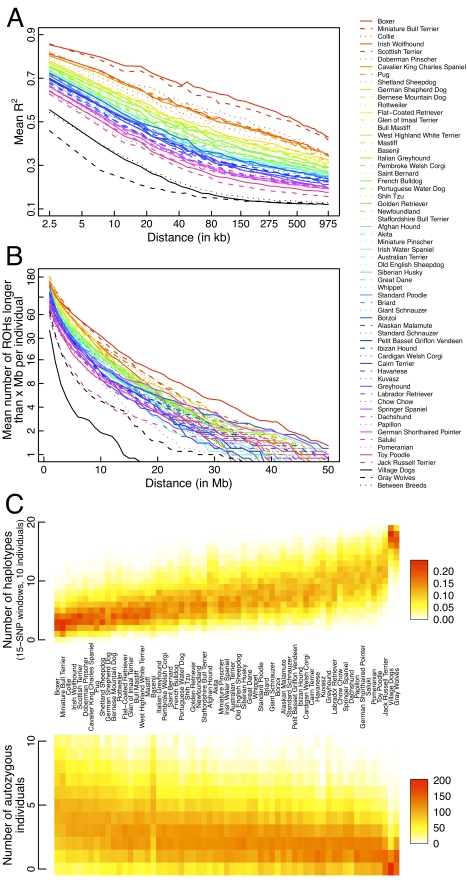
Analysis of 10 individuals from each of 59 breeds, one population of village dogs, and wolves. (A) LD decay curves based on mean r^2^, including mean LD decay when dogs are selected from 10 different breeds (“between breed” LD). (B) Distribution of long runs of homozygosity in each group. (C) Number of haplotypes across all autosomal 15-SNP windows and number of autozygous individuals per breed at each genomic position computed using 10 individuals per breed. Each window can contain 1–20 different haplotypes and each genomic position can have 0–10 individuals appearing autozygous.

We find that while LD extends over 1 Mb within every breed surveyed, across all dogs combined it decays extremely rapidly, consistent with previous studies [4],[15]. This suggests few IBD segments are shared across multiple breeds and those that are shared are quite small (Figure 1A). Homozygous runs are also longer and more numerous in breed dogs than village dogs or wolves (Figure 1B), with individuals from nearly every breed exhibiting 10–50 ROHs greater than 10 Mb. Interestingly, Jack Russel terriers are an extreme outlier, with fewer such ROHs and, overall, higher levels of diversity than other dog breeds. Autozygosity levels were high in all breeds (lowest mean autozygosity = 7.5% in Jack Russel terriers, highest mean autozygosity = 51% in boxers); however, very few breeds exhibited genomic regions that were autozygous in all individuals at the megabase scale. In contrast to human populations, where patterns of autozygosity in populations are not generally correlated with haplotype diversity [16], pure bred dogs show a very strong negative correlation between autozygosity and haplotype diversity (Figure 1C). One notable exception is basenjis, which show high haplotype diversity and high autozygosity, suggesting a recent population bottleneck post-breed formation has induced higher levels of inbreeding than expected. This is consistent with the breed history of basenjis in the United States, which are believed to descend from a small founder population.

It has previously been shown that LD extends much further within breeds than it does among breeds or within wolves [17],[18]. Our analysis reveals that between-breed LD is significantly greater than wolf LD, consistent with a bottleneck in dogs during domestication. LD within the single village dog population decayed at a similar rate as LD between dog breeds, also consistent with a shared domestication bottleneck shaping LD patterns in both breed and village dogs. Perhaps surprisingly, village dogs exhibited fewer long ROHs than wolves, indicating that, at least in historical times, village dogs have likely maintained a higher effective population size or better inbreeding avoidance than their gray wolf progenitors. Similarly, haplotype diversity was also marginally higher in village dogs than in gray wolves across 500 kb windows (Figure 1C). Taken together, these observations suggest a radical reshaping of the dog genome on multiple time scales with the recent process of breed formation playing a particularly important role in transforming ancestral genetic variation into differences among breeds that show a high degree of genetic and phenotypic uniformity.

### Genome-Wide Scan for Recent Selection

**Figure 2:**
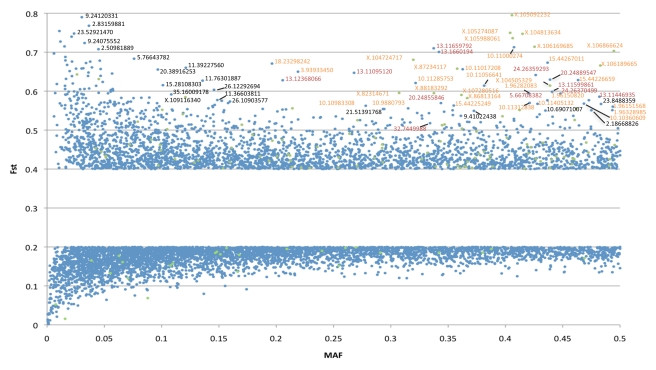
F_ST_ for each SNP across the 79 CanMap breeds. Red indicates SNPs with known associations to morphological traits (dark red to fur traits). Mean F_ST_ was 0.28 (SNPs with F_ST_s between 0.2 and 0.4 are not plotted here).

Given our finding of little sharing of IBD segments among individuals from different breeds, we expect that when coincident sharing occurs across breeds with a similar phenotypic trait, these genomic segments likely underlie heritable variation for that trait. We searched for the strongest signals of allelic sharing by scanning for extreme values of Wright's population differentiation statistic F_ST_ [19],[20] across the breeds. The 11 most extreme F_ST_ regions of the dog genome contained SNPs with F_ST_≥0.57 and minor allele frequency (MAF)≥0.15 (Table 1). Six of these regions are strongly linked to genetic variants known to affect canine morphology: the 167 bp insertion in *RSPO2* associated with the fur growth and texture [8], an *IGF1* haplotype associated with reduced body size [2], an inserted retrogene (*fgf4*) associated with short-leggedness [5], and three genes known to affect coat color in dogs (*ASIP*, *MC1R*, and *MITF* [4],[21],[22]). Two other high F_ST_ regions correspond to CFA10.11465975 and CFA1.97045173, which were associated with body weight and snout proportions, respectively, in previous association studies [23],[24]. Two known coat phenotypes (fur length and fur curl [8]) also exhibited extreme F_ST_ values. Only a limited number of high F_ST_ regions were not associated with a known morphological trait (Figure 2, black labels). Here, we focus on illuminating the potential targets of selection for these regions as well as identifying genomic regions that associate with skeletal and skull morphology differences among breeds.

**Table 1:** Summary of SNPs with F_ST_>0.55 and minor allele frequency (MAF) >15% across CanMap breeds.

		Derived Allele Frequency					
Marker	F^ST^	Dog	Wolf	Coyote	Jackal	F_ST_ Region	Trait
X.105092232	0.795	0.594	1.000	0.000	0.000	1045486877–108201633	body size; skull shape
10.11000274	0.713	0.593	0.031	0.000	0.000	10707312–11616330	ear type [23]; body size [7],[23],[47]
13.11659792	0.710	0.337	0.000	0.000	0.000	11659792–11660194	furnishings[8]
15.44267011	0.673	0.437	0.008	0.000	0.000	44187156–44427593	body size[[2],[23],[47]]
18.23298242	0.671	0.196	0.287	0.042	0.778	singleton	height [5]
X.87234117	0.658	0.642	0.505	0.000	0.267	86813164–87299370	limb/tail length
3.93933450	0.650	0.219	0.111	0.000	0.250	singleton	body size
24.26359293	0.641	0.426	0.000	0.000	0.000	26359293–26370499	coat color[4]
20.24889547	0.630	0.561	0.382	0.286	0.000	24674148–24969549	coat color[22]
1.96282083	0.594	0.580	0.227	0.000	0.667	96103038–96338823	snout ratio[23]
5.66708382	0.576	0.437	0.016	0.000	0.000	singleton	coat color[21]
1.71150281	0.573	0.160	0.177	0.000	0.000	71150281–71206706	
26.10903577	0.569	0.158	0.000	0.000	0.000	singleton	
23.8488359	0.567	0.483	0.024	0.250	0.000	singleton	
1.59179746	0.554	0.188	0.629	0.550	0.000	59179746–59182125	snout length
21.51391768	0.554	0.293	0.414	0.929	0.000	singleton	
15.32610857	0.554	0.294	0.009	0.000	0.000	32383555–33021330	
1.114924791	0.553	0.209	0.000	0.000	0.000	114914236–114924791	
29.30499875	0.553	0.205	0.359	0.000	0.000	singleton	
16.55231367	0.551	0.155	0.145	0.125	0.000	singleton	
2.18668826	0.551	0.475	0.066	0.000	0.000	singleton	
10.69071007	0.550	0.435	0.140	0.500	0.000	69071007–69166227	

Derived allele determined by the minor allele in jackals (black-backed and side-striped) and coyotes. Each F_ST_ region is defined as the genomic region surrounding the top F_ST_ hit where neighboring SNPs on the array also had F_ST_s above the 95th percentile (F_ST_ = 0.4). Traits with associations to each region are listed; underlining denotes an association from this study.

### Genome-Wide Association Mapping of Morphological Differences among Dog Breeds

We investigated the genetic architecture of morphological variation in dogs using a breed-mapping approach to look for correlations between allele frequency and average phenotypic values across 80 breeds at 60,968 SNPs (see Methods). We computed male breed-average phenotypes for each of 20 different tape measurements, and also computed breed averages from museum specimens for 15 long bone and 20 skull/tooth dimensions (Figure S1). For all 55 measures, we conducted association scans with and without controlling for overall breed body size, and also controlled for breed relatedness by using breed-average relatedness as a random effect in the linear mixed model. We also looked for genomic regions underlying body size variation and ear floppiness.

**Figure 3:**
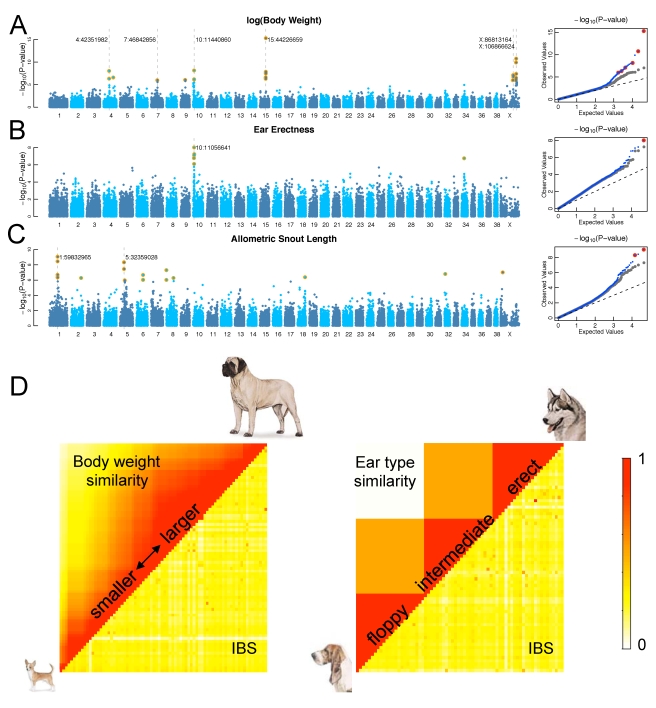
Genome-wide association scans across the breeds using allele frequencies of the SNPs and breed-average phenotypes for (A) log(body weight), (B) ear erectness (floppy versus erect ears), and (C) allometric snout length. The *p* values of the SNPs were computed using the linear mixed model method for (A and C) and weighted permutation method for (B). SNPs passing Bonferroni correction are marked with orange circles; SNPs included in best-fit predictive models are marked with gray dashes. P-P plots for the scans are shown in the right-hand column. (D) Matrix showing phenotype identity (upper diagonal) is uncorrelated with genome-wide IBS (lower diagonal) between dog breeds for body weight and ear type. Genome-wide IBS is plotted as a scaled value where 0 corresponds to the lowest amount of IBS between any two breeds (0.62) and 1 corresponds to the highest amount of IBS (0.83). Boxers are not shown since their IBS values are low in comparison to other breeds due to the SNP ascertainment bias on the array.

Body size variation is greater across dog breeds than in any other terrestrial species [25], with smaller stature likely being selected for during domestication and with large and small body sizes being alternatively selected for in different breeds. Our scan for body size (defined as log(body weight)) yielded several significant genomic associations, with the six strongest signals occurring at CFA15.44226659, CFAX.106866624, CFA10.11440860, CFAX.86813164, CFA4.42351982, and CFA7.46842856. The corresponding P-P plot compares the observed distribution of −log_10_ *p* values (i.e., blue and red points in Figure 3A) to the expected distribution under a model of no-association (i.e., dashed line which represents equality of Expected and Observed) and demonstrates an excess of significant signals since the tail of the distribution is well above the diagonal dashed line. When the top six regions (and linked SNPs) are removed, the observed *p* value distribution (i.e., gray points in Figure 3A) is strongly shifted towards the null expectation, suggesting these six QTLs account for the bulk of the association signal in our data. The first four signals are among the highest F_ST_ regions in the dog genome (Table 1) with the CFA4 signal also exhibiting an elevated F_ST_ (0.46), consistent with diversifying selection among breeds for body size. The signal on CFA15 corresponds to the location of *IGF1* which encodes a growth factor previously described to control a significant proportion of size variation across dog breeds [2]. The CFA10 signal corresponds to the location of *HMGA2*, a gene known to affect body size variation in humans [26] and mice [27]. Both *HMGA2* and a locus corresponding to the CFA7 signal, *SMAD2*, have been previously associated with dog body size [23]. In contrast, the signals on CFA4 and CFAX hits have not previously been associated with body size variation in dogs. Interestingly, the CFA4 signal contains (among other genes) the *STC2* locus, a known growth inhibitor in mice [28]. The two signals on the X chromosome lie in separate LD blocks that each contains dozens of genes. Other than *IGF1*, all the other regions will require fine-mapping in order to confirm a single candidate gene. In all six regions, wolves are not highly polymorphic (MAF<0.1), and except for the CFA10 signal, the derived allele is at highest frequency in small breeds.

Another key trait that varies substantially among breeds is ear type. All adult wild canids have erect ears, but dog breeds are alternately fixed for various ear positions, including floppy ears. This paedomorphic trait is retained by adults of some breeds in many domesticated mammals, including dogs, cattle, goats, and rabbits. We looked for SNPs associated with breeds fixed for floppy or erect ears, and found a single region on CFA10 that is likely responsible for the difference in ear type (Figure 3B). The derived allele at this locus is nearly fixed in floppy-eared breeds, consistent with the floppy ear position being the derived phenotype (Figure S2). This SNP is also within a region associated with body size in this study (near *HMGA2*), although the strongest signal for ear position occurs nearly 0.5 Mb upstream, near *MSRB3* (Figure S3). Floppy-eared breeds show sharply reduced heterozygosity, suggesting this region, the highest F_ST_ region in any autosomal segment of the dog genome (Table 1), has undergone strong selection for floppy ear position or perhaps some correlated trait.

Snout length is another trait that varies considerably among breeds, and like floppy ears, short snouts are associated with neoteny in many domesticated mammals [29]. Association mapping using breed-average values for absolute snout length highlights similar genetic regions as those suggested for body weight, but introducing log(body weight) as a covariate in the association model allows for an allometric correction and reveals QTLs underlying proportional snout length (Figure 3C). The strongest signals for proportional snout length are CFA1.59832965 and CF5.32359028. Both are within the top 5% of high F_ST_ SNPs (F_ST_ = 0.55 and 0.42, respectively) and are only found at high derived-allele frequency in breeds with short snouts (brachycephalic).

**Figure 4:**
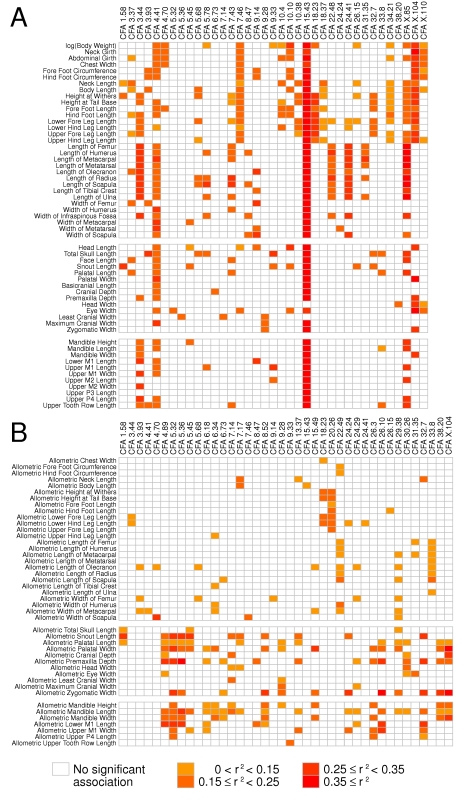
Summary of associations across genomic regions for multiple traits. Each row corresponds to a trait (either absolute or proportional), and each column corresponds to a genomic region that has been found associated with at least one trait. The shading of each rectangle shows the R^2^ statistic of the single marker model for the trait for all significant associations (*p*<5.0e-5 for absolute external traits, *p*<1.0e-4 for skeletal and proportional traits after correcting for population structure). When multiple SNPs in the region are significant, the largest value of the R^2^ statistics is reported.

Using forward stepwise regression, we combined potential signals into a multi-SNP predictive model for each trait. In the models of body weight, ear type, and the majority of measured traits, most of the variance across breeds could typically be accounted for with three or fewer loci (Figure 4 and Table S1). Correlated traits (e.g., femur length and humerus length) yielded similar SNP associations. For the 55 traits, the mean proportion of variance explained by the top 1-, 2-, and 3-SNP models was R^2^ = 0.52, 0.63, and 0.67, respectively (see Table S1). After controlling for body size, mean proportion of variance explained by these models was still appreciable—R^2^ = 0.21, 0.32, and 0.4, respectively. Notably, the most significant genomic regions were similar even using naïve association scans that did not control for population structure (Figure S4). In terms of breed mapping, the level of population structure common to groups of breeds was insufficient to strongly bias association inferences (see Figure S5 and Methods).

For most of the traits investigated, we found that a few QTLs of large effect governed the phenotypic differences among breeds. For example, for proportional height at withers, we observe a large-effect QTL on CFA18 that we have previously shown corresponds to an *fgf4* retrogene that accounts for chrondrodysplasia or disproportional dwarfism in breeds such as corgis, basset hounds, and dachshunds [5], although we also find a novel QTL for height on CFA20. Likewise, skull shapes were largely dictated by regions on CFA1, CFA5, CFA26, and CFA32. In addition, the CFAX.105274087–106866624 region that was associated with body size is also associated with skull length, even after accounting for breed-average body weight. Nearly all of these regions were also associated with dental traits, in addition to a strong association on CFA16, suggesting a suite of correlated traits that are principally governed by a few genomic regions.

### Validation

**Figure 5:**
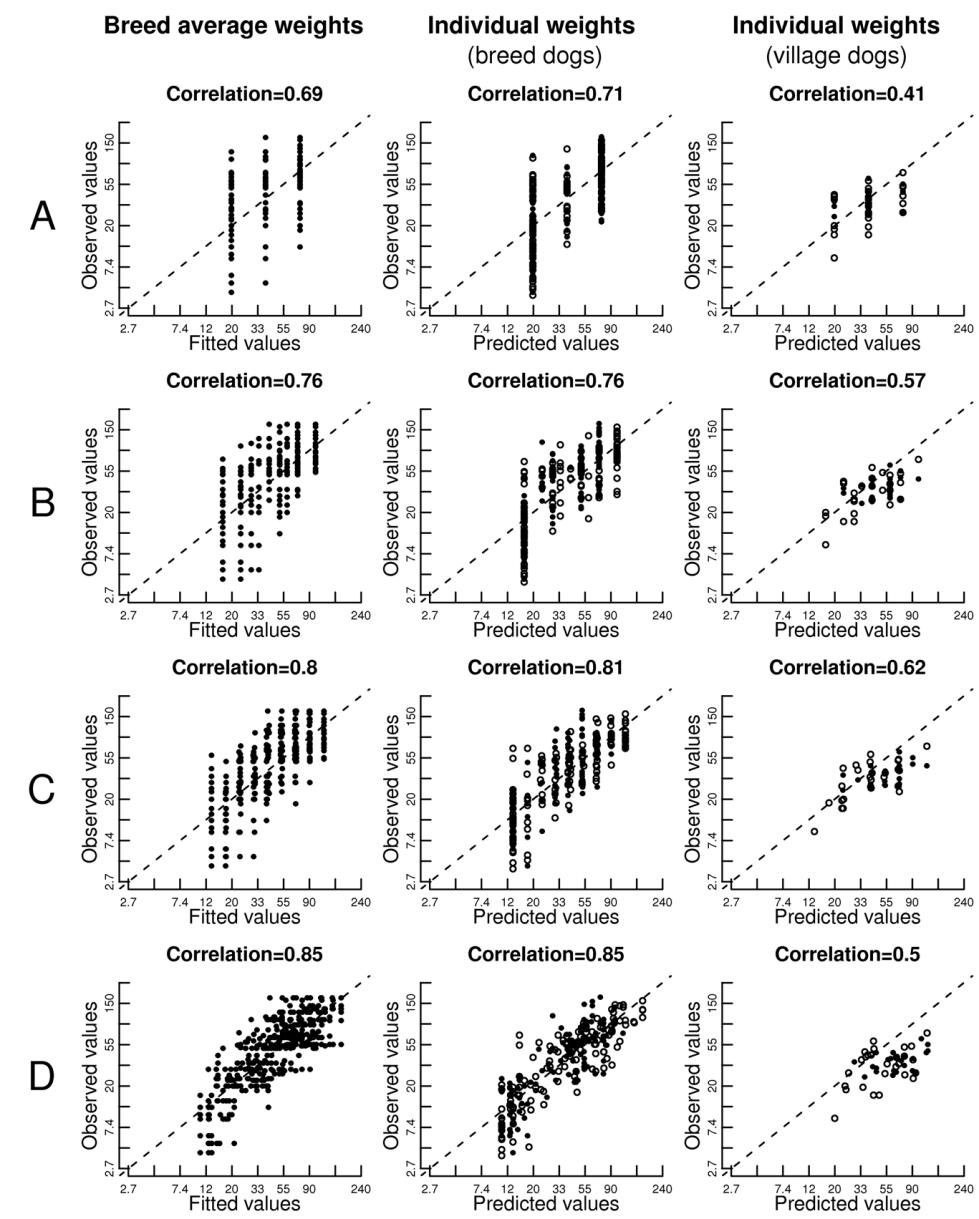
Correlation between observed and predicted log(body weight) using regression models based on breed-average data. Plots show correlation with observed breed-average values (1st column), 249 individually phenotyped breed dogs (2nd column), and 50 non-breed village dogs with individual measurements. (A) The predictive model using a single SNP, CFA15.44226659; (B–D) the predictive models using 2, 3, and 6 top SNPs (in order after CFA15.44226659, CFAX.106866624, CFA4.42351982, CFAX.86813164, CFA10.11440860, and CFA7.46842856).

To test whether the SNPs that account for differences among breeds also account for among-individual variation, we used a cross-validation approach. For example, we used the top six SNPs associated with breed-average body weight to compute the best-fitting linear predictor of body size, while ignoring epistasis and non-additive effects at the individual level. We validated this model using two populations with known individual weights: 249 dogs from breeds included in CanMap and 50 previously measured outbred village and shelter dogs from Africa and Puerto Rico [13] that were genotyped at the top six body-weight-associated loci. The linear model explained the majority of body size variation in both the breed dogs and the non-breed village dogs (correlation coefficients of 0.85 and 0.50, respectively; see Figure 5). Most of the variance in body size was explained by the *IGF1* locus where we observe a single marker with R^2^ = 50% and R^2^ = 17% of variance in breed and village dogs, respectively. The top 3-SNPs explain R^2^ = 38% of the variance in body weight in village dogs, although the 6-SNP model explains less. The lower R^2^ in non-breed dogs than breed dogs may be a consequence of lower LD observed in village dogs reducing the strength of association between these markers and the causal body size variants. Alternatively, the lower R^2^ may also be a consequence of non-genetic factors such as diet or measurement error affecting the observed village dog weights, the smaller range of body sizes observed in the non-breed dog sample, or perhaps to overfitting of the model based on the particular breeds included in the dataset. Nevertheless, R^2^ = 38% is significantly better than association scans for morphometric traits in humans utilizing denser marker arrays (e.g., [30]), suggesting that, at least for some quantitative traits like body size, both the initial dog domestication event(s) and the subsequent artificial selection in closed breed populations are responsible for simplifying the underlying genetic architecture of trait variation.

## Discussion

Written into the genome of modern domestic dogs are the genetic footprints of the demographic and selective forces underlying their transition from ancestral gray wolves. Patterns of LD demonstrate a bottleneck at domestication that is shared by village and breed dogs but not wolves. This was followed by occasionally strong breed-specific bottlenecks. The strong artificial selection and drift within essentially independent breed populations allows for the efficient detection of significant genetic associations with quantitative traits which, at least for body size, also seem to account for phenotypic variation within outbred village dogs. Regions associated with morphological variation account for at least the 11 top F_ST_ regions identified across dog breeds, consistent with both strong selection for morphology and a simplified genetic architecture for these quantitative traits in dogs. Genomic analysis of other village dog and gray wolf populations and additional phenotyping will no doubt further enrich our understanding of the process of domestication and artificial selection in dogs.

In humans, high-F_ST_ regions are associated with hair and pigmentation phenotypes, disease resistance, and metabolic adaptations [31]. In contrast, the strongest signals of diversifying selection in dogs are all associated with either body size/shape or hair/pigmentation traits, and therefore are unlikely to have been under selection for disease resistance, metabolic adaptations, or behavior. In total, the 11 highest F_ST_ regions identified across purebred dogs are all associated with body size/shape or hair phenotypes, including three genomic regions that had not been detected in previous association studies.

Our association scans offer a sharp contrast to recent findings on the genetics of quantitative traits in humans such as height, weight, BMI, and blood pressure, as well as susceptibility to a litany of metabolic and cardiovascular disorders [30],[32]. For example, genome-wide association studies in humans using tens or hundreds of thousands of samples and ≥500,000 SNPs suggest that most phenotypic variation in our species is governed by a large number of variants of small effect [33]. In contrast, often only two to six QTLs are needed to explain large amounts (often >70%) of the variance in most of the morphological traits we studied across domestic dog breeds. A similar pattern of few QTLs of large effect is apparent in a few other genetic systems (e.g., sticklebacks [34]), suggesting this genetic architecture could be a result of recent adaptation and a hallmark of diversifying selection.

The dominance of a few genes of large effect likely reflects several unique aspects of selection in dogs. First, many of the modern breeds were created during the Victorian Era where novelty was a focus of selection and breeders favored the preservation of discrete mutations. A single discrete mutation could be placed on a variety of genetic backgrounds by crossing which expanded the range of phenotypic diversity across breeds. For example, the same retrogene responsible for chondrodysplasia or foreshortened limbs (*fgf4*) is found in nearly 20 distinct breeds today [5]. In contrast, the progressive selection in other domestic species aimed at economically useful quantitative traits such as a high growth rate and fecundity involved subtle differences among individuals selected across many generations and, therefore, likely utilized genes of small effect segregating in an ancestral population [11]. Mutations of large effect are the basis of some domesticated phenotypes, such as the double-muscling gene in cattle [35], but the selective breeding practiced for agriculture was more intensive and sustained and drew on a segregating variation that involved the detection of small differences among individuals.

Selection for novelty also led to extreme founder events and/or bottlenecks in many breeds. Coupled with the dog domestication bottleneck, this likely simplified the genetic architecture of quantitative traits, including complex disease phenotypes that are not fixed within breeds and were not the subject of selection for novelty. The rapid genetic drift between isolated breeds (pairwise F_ST_ of 25%–30% among any given set of breeds with very few pairs of breeds having significantly smaller F_ST_) enables efficient mapping of the genomic regions underlying variation, even in some cases with un-genotyped collections such as museum specimens. The extreme phenotypic diversity observed among modern domestic dogs is unique among mammalian species, and as such, it offers unique insight regarding both the constraints and potential of evolutionary change under domestication.

## Methods

### SNP Calling

We genotyped 1,659 samples on Affymetrix v2 Canine arrays which contain probes for over 127,000 SNP markers, and attempted to call genotypes on the 1,400 arrays with highest signal-to-noise intensity ratios. SNP content for this array includes variants found from the boxer genome assembly (25.5% of SNPs), comparison of the boxer and poodle assemblies (11.4% of SNPs), comparison of the boxer to low coverage sequencing from other breeds (59.9% of SNPs), and comparison between dog and wolf sequences (3.2% of SNPs). Similar to previous studies [4], we found that the BRLMM-P algorithm yielded approximately 45,000 SNPs (out of 127K markers present on the array) that passed quality control filtering, and that it consistently over-called heterozygous genotypes. Consequently, we developed a novel genotype calling algorithm, MAGIC (Multidimensional Analysis for Genotype Intensity Clustering), which did not use prior information regarding cluster locations, assumptions about Hardy-Weinberg equilibrium, or complex normalization of probe intensities (see Text S1 and Tables S2 and S3). On these same 1,400 chips, MAGIC called 60,968 SNPs that passed our quality control filters, yielding a call rate of 94.6% and a concordance rate of over 99.9% for samples run in duplicate. Over 99% of SNPs used in our analysis are within 121 kb of another SNP (median = 8.5 kb).

As a final quality control step, we applied the hidden Markov model described in [16] to detect genomic regions of autozygosity within each of the 1,400 CanMap individuals. Since mean autozygosity was above 20% in the dataset, we expect nearly 300 individuals to be within an autozygous segment at any SNP on the array. All of these ∼300 individuals should have homozygous genotype calls for that SNP, although in practice some heterozygous calls can be expected owing to gene conversion or imperfect inference of the autozygous segments. SNPs with poor genotyping quality, specifically those SNPs with a spurious excess of heterozygous calls, will exhibit relatively high rates of heterozyosity even within inferred segments of autozygosity. We excluded 451 SNPs with elevated heterozygosity within autozygous segments (here defined as >10%). Visual inspection of the cluster plots suggests many of these SNPs occurred within segmental duplications or copy number variable regions, or contained a substantial fraction of null alleles mistakenly called as heterozygous.

### LD Decay

We summarized pairwise LD by the genotype correlation coefficient (*r* ^2^). For all pairs of autosomal SNPs, *r* ^2^ was calculated using the --r2 --ld-window 99999 --ld-window-r2 0 command in PLINK. Since we calculated *r* ^2^ using the genotypes directly without phasing the data, this analysis is robust to phasing ambiguities.

To compare LD decay among breeds with different sample sizes, we selected a random subset of 10 dogs from each of the 59 breeds having 10 or more dogs. Within each breed, we calculated *r* ^2^ between all pairs of SNPs where both SNPs had MAF≥15% and <10% missing data. Thus, different pairs of SNPs were used for different breeds, with the number of SNP pairs ranging from 147,082 to 321,899.

### Phasing

We inferred haplotype phase using the program fastPHASE version 1.4.0 [14]. We phased all individuals together in a single run but designated dogs from different breeds as members of different subpopulations. This procedure was shown to yield optimal results when phasing human data [36]. We specified the number of haplotype clusters (*K*) to be equal to 40. Through preliminary runs using subsets of the data, we found that the genotype imputation error rate (as estimated from masking and imputing known genotypes) continues to decrease as K increases (up to *K* = 100), albeit slowly. This suggests that higher values of *K* may yield more accurate results. However, since the practical advantages of using higher values of *K* were not clear, we assessed the sensitivity of the number of haplotypes per breed to the value of *K* used. We found that the value of *K* had little impact on the overall results, and thus chose *K* = 40 as a compromise between the true number of “haplotype clusters” in the sample and computational efficiency. We included 49,508 SNPs in the phased haplotypes that had MAF≥1% and <10% missing data in the entire sample of dogs.

### Haplotype Diversity

To summarize haplotype diversity within each dog breed, we used the number of distinct haplotypes within each window in windows across the genome. This statistic has been shown through simulations and empirical data to be informative regarding population history [16],[37]. Since the number of SNPs within each window is a complex function of the mutation rate, evolutionary stochasticity, and the ascertainment process, we did not want our measure of haplotype diversity to be influenced by the number of SNPs within a window. Therefore, we divided the genome into 500 kb windows and selected a random subset of 15 SNPs from all windows with ≥15 SNPs. For windows with <15 SNPs but at least 5 SNPs, we selected 5 SNPs at random. Windows with fewer than 5 SNPs were dropped from the analysis. The same randomly selected SNPs were used for all breeds. We then counted the number of distinct haplotypes within each breed for each window using the inferred haplotypes from fastPHASE. Since the number of haplotypes is influenced by the sample size, we selected a random subset of 10 dogs from each breed for this analysis.

### Autozygosity

To detect runs of homozygous genotype calls indicative of autozygous segments, we implemented the hidden Markov model described in [16] which has been shown to be robust to marker ascertainment bias. We assumed a recombination rate of 1.0 cM/Mb, a genotyping error rate of 0.5%, and prior probabilities of autozygosity and non-autozygosity of 20% and 80%, respectively. All other parameters were as in [16]. Using a forward-backward algorithm, we found all putative runs of autozygosity >100 kb spanning at least 25 SNPs.

### Genome-Wide Scans

#### Phenotypic values

The traits we investigated here include body weight, external measurements (e.g., height at withers, body length, etc.), and bone measurements (skull and skeleton measurements). Since these measurements are not available for most of the genotyped samples in the CanMap dataset, we treated breed averages as breed characteristics and assigned them to each individual of the same breed as phenotypic values as has been suggested previously [23]. The breed averages of body weight were obtained from [38]. The breed averages of external measurements were computed from questionnaire data, provided by dog owners, and contain 58 breeds that have genotyped individuals in the CanMap dataset. Using dogs older than 1 y, we computed the breed average of each trait for which at least two individuals had been measured. The breed averages of bone measurements were computed from the samples described in [39].

We used the breed averages of the traits for model selection, i.e., to prioritize SNPs for association, model fitting, and to fit the predictive model using associated SNPs. To account for allometry, we included log(body weight) as a covariate in the model. Some samples in the CanMap dataset also had individual body weights or external measurements. These data were used for the purpose of model validation.

#### Genome-wide scans for associations

All the SNPs that passed the quality filtering were considered in the scans for association. The allele frequencies were computed for each breed for all SNPs. An individual-by-individual IBS similarity matrix was calculated and then averaged within breeds to obtain a breed-average IBS matrix, which was used to control for genetic relatedness among breeds.

For continuous traits, a linear mixed model [40],[41], as implemented in EMMA [42], was used to test each of the SNPs for association while also controlling for relatedness. Here, because mapping is being done at the breed level, we used EMMA to control for relatedness between (rather than within) breeds. The random effects were assumed to follow a multivariate normal distribution with a mean of 0 and the correlation matrix being the breed average IBS matrix [42]. For allometric traits, we used log(breed average body weight) as a covariate in the linear mixed model for all traits except for those skull- and tooth-related skeletal traits, for which we used log(breed average total skull length).

For dichotomous traits, a weighted bootstrap method was used to test each of the SNPs for association. The phenotypes were bootstrapped with weights accounting for breed relatedness, and the empirical distributions of test statistics were obtained for calculating *p* values. Each round of bootstrap consisted of *N* steps where the sample size was *N*. The IBS matrix was denoted as *K* with the value between breed *i* and breed *j* equal to 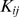. At step *i*, we sampled a phenotype for the *i*th individual from the *j*th individual, where *j* is chosen with probabilities proportional to row *i* of the IBS matrix. Specifically, we chose the phenotype corresponding to individual *j* with probability 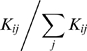. A 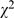 correlation test-statistic was obtained for each round of bootstrapped phenotype and the SNP breed frequencies. The empirical *p* value was the number of bootstrap replicates that showed the test-statistic bigger than the test-statistic obtained from the observed phenotype. For all the scans, naïve tests without accounting for breed relatedness were also employed for comparison.

#### Model fitting and validation

We use the results of single marker EMMA scans described above in constructing multi-SNP models for predicting phenotype from genotype. Specifically, we use forward stepwise regression with breed average value of the trait as the dependent variable and a design matrix consisting of individual dog genotypes across the most highly associated SNPs from the EMMA breed-level scan. For those traits with individual phenotype and genotype measurements (such as body weight), we used the multi-SNP predictive models for validation. Specifically, for all individuals with both genotype and phenotype data, we predicted an individual's phenotype by applying the multi-SNP model to their individual genotype data and compared the observed and predicted values. The predictive models for body weight were also validated on a dataset of 50 village dogs with individual body weights and genotypes across the associated SNPs [13].

### Analysis of Population Structure in Breed Dogs Using Structure and PCA

A potential confounding factor in our study is relatedness among breeds that share traits of interest. For example, if small dog breeds are more closely related, on average, to each other than large dog breeds, then the loci identified may simply be distinguishing genomic regions associated with historical relatedness (and not size, per se). To test this notion, we undertook a systematic dissection of the population structure of modern dog breeds. Using 5,157 unlinked SNPs genotyped on 890 dogs from 80 breeds, we evaluated population structure using Principal Component Analysis (PCA; [43]) and the Bayesian clustering program Structure (Figure S4) [44],[45]. Both methods distinguish “ancient” and “modern” breeds in their initial clustering (*K* = 2 or PC1) as previously observed with boxers (one of the two main breeds used for SNP ascertainment) and basenjis (the only African breed in the sample) being identified next (*K* = 3,4 or PC2/3). Importantly, in both methods breed groups did not tend to form clusters; instead, single breeds or pairs of closely related breeds are “pulled out” as one examines higher dimension PCs or adds new Structure groups (i.e., increases *K*). When Structure was run at *K* = 80, three pairs of breeds and one trio were indistinguishable (Samoyeds – American Eskimo Dogs, Collies – Shetland Sheepdogs, Bull Terriers – Miniature Bull Terriers, and Chow Chows – Akitas – Shar Pei) and some of the 80 clusters became degenerate, as has been reported previously with cluster analysis using microsatellites [17],[46]. However these breeds were still separated out by PCA (for example, PC29 separates Chows and Akitas, PC42 separates Shetland Sheepdogs and Collies, etc.). This pattern was consistent with modern breeds being, for the most part, a recent adaptive radiation (star phylogeny) with few significant internal branches. In fact, a Molecular Analysis of Variance suggested only 4% of the genetic variance was attributable to major phenotypic groupings (such as herding/gun/toy, see also [12]).

## Supporting Information

Figure S1 **Diagrams depicting a subset of measurements used to calculate breed averages for morphological trait mapping.** (A) Body measurements taken on live dogs. Red lines represent the path of superficial measurements. The skeleton is shown for anatomical clarity. Measurements collected included: height at withers (1), height at base of tail (2), snout length (5), head length (6), neck length (7), body length (8), tail length (9), neck girth (11), abdominal girth (12), hind foot length (14), hind foot circumference (15), lower hind leg length (16), upper hind leg length (17), fore foot length (18), fore foot circumference (19), lower fore leg length (20), upper fore leg length (21). (B) The skull measurements taken on the museum specimens. The measurements include: total skull length (TSL), face length (FL), upper tooth row length (TRL), upper P3 length (P^3^L), upper P4 length (P^4^L), upper M1 length (M^1^L), upper M2 length (M^2^L), maximum cranial width (MCW), zygomatic width (ZW), least cranial width (LCW), cranial depth (CD), premaxilla depth (PD), mandible height (MH), mandible length (ML), lower M1 length (M_1_L), basicranial length (BCL). The cranioskeletal diagram was reproduced with author permission from Wayne, R. (Evolution 40, 243–261, 1986).
(0.45 MB PDF)

Figure S2 **Correlation between the allele frequency of the most highly associated SNP (lower diagonal) and the phenotype for (A) log(body weight) and (B) ear floppiness (upper diagonal) across the 80 CanMap breeds.**
(0.24 MB PPT)

Figure S3 **Fine-scale resolution of CFA10 region associated with both body size traits and ear floppiness.** Single-marker analysis shows strongest association with body weight near *HMGA2*, while the strongest association with ear floppiness is near *MSRB3*. High F_ST_ between small- and large-breed dogs and reduced heterozygosity in small breed dogs extends several hundred kb away from *HMGA2*. The strongest ear flop association and F_ST_ signal between erect- and floppy-eared breeds are relatively localized within 100 kb region near *MSRB3*, although reduced heterozygosity in floppy-eared breeds extends for 500 kb.
(0.49 MB PDF)

Figure S4 **Genome-wide association scans using naïve tests without accounting for breed relatedness.** Scans show (A) log(body weight), (B) ear erectness (floppy versus erect ears), (C) proportional snout length, (D) proportional palatal length, and (E) snout type (brachycephalic versus average).
(0.30 MB PPT)

Figure S5 **Population structure across CanMap breeds determined by PCA (top) and Structure (bottom).** Each individual is a thin column and individuals are grouped by breed (black vertical lines separate breeds, with bold lines denoting separation between breed groups). Values for PC1 through PC80 are shown in descending order for each individual by color with blue indicating lower-than-average PC values and red indicating higher-than-average values. The height of each PC is proportional to the proportion of variance explained by each principal component (shown on right axis). Ordering of individuals along the *x*-axis (6–12 individuals per breed) is identical for both panels.
(0.47 MB PDF)

Table S1 **Proportion variance explained by models incorporating the top one to six SNPs for each trait.** Blanks denote traits with too few significant SNPs to parameterize a full model.
(0.10 MB DOC)

Table S2 **Comparison of BRLMM-P and MAGIC genotype calling algorithms using common Affymetrix .cel files and QC filters.** Note that the 1,400 arrays used for the analyses in this study are a subset of the arrays used to conduct this head-to-head comparison, so total SNP counts differ somewhat between the datasets.
(0.05 MB DOC)

Table S3 **List of SNPs that were sequenced to validate the MAGIC genotyping algorithm.** Red SNPs indicate discordant homozygous calls between MAGIC and BRLMM, which are indicative of the presence of “null alleles” (individuals lacking specific binding to either probe, usually because of a variant at the probe binding site).
(0.06 MB DOC)

Text S1 **Algorithmic details and validation of MAGIC genotype calling program.**
(0.12 MB DOC)

## Abbreviations

AKC: American Kennel Club
BMI: body mass index
LD: linkage disequilibrium
MAF: minor allele frequency
QTL: Quantitative Trait Loci
