# Supplementary figures and images for: A Simple Genetic Architecture Underlies Morphological Variation in Dogs
Source: PLoS Biol. 2010 Aug 10;8(8):e1000451. doi: 10.1371/journal.pbio.1000451 (PMC2919785; doi:10.1371/journal.pbio.1000451)

A

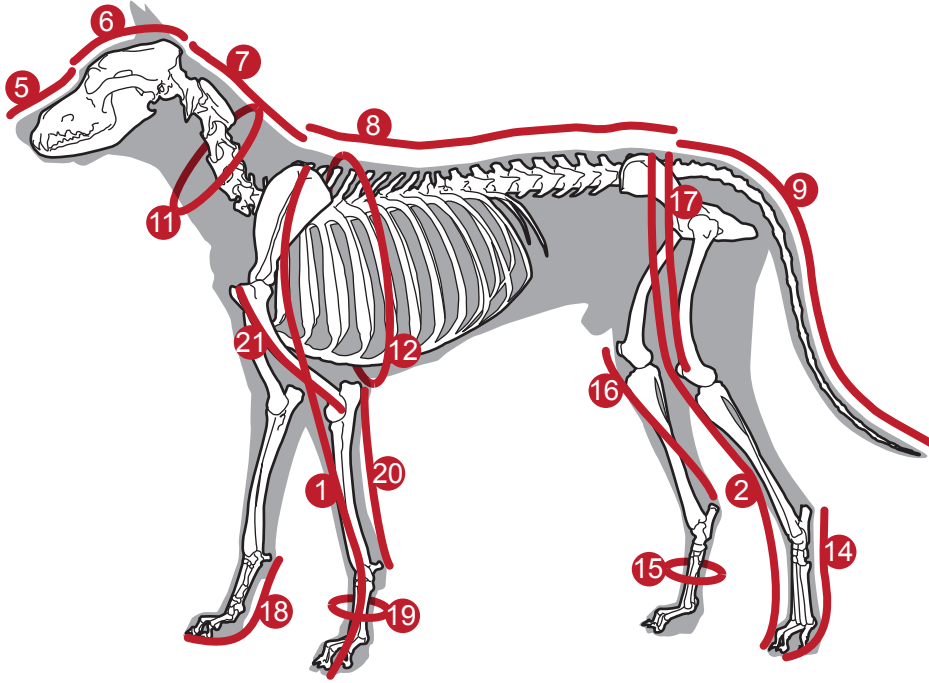

B

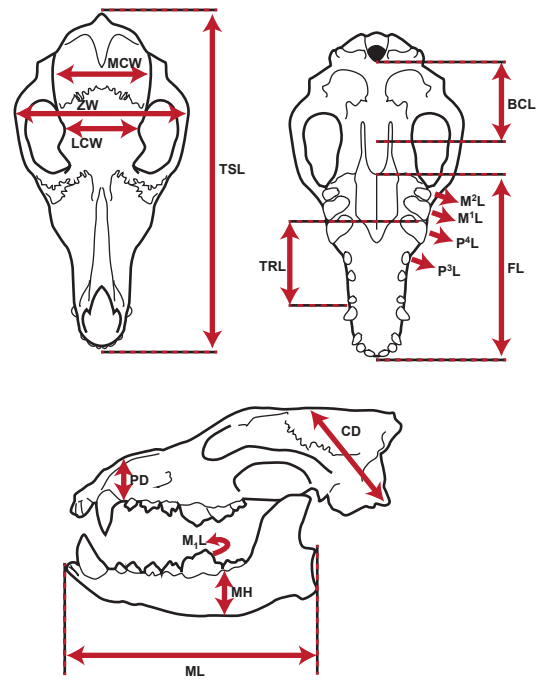

Supplement: Figure S1 — Diagrams depicting a subset of measurements used to calculate breed averages for morphological trait mapping. (A) Body measurements taken on live dogs. Red lines represent the path of superficial measurements. The skeleton is shown for anatomical clarity. Measurements collected included: height at withers (1), height at base of tail (2), snout length (5), head length (6), neck length (7), body length (8), tail length (9), neck girth (11), abdominal girth (12), hind foot length (14), hind foot circumference (15), lower hind leg length (16), upper hind leg length (17), fore foot length (18), fore foot circumference (19), lower fore leg length (20), upper fore leg length (21). (B) The skull measurements taken on the museum specimens. The measurements include: total skull length (TSL), face length (FL), upper tooth row length (TRL), upper P3 length (P3L), upper P4 length (P4L), upper M1 length (M1L), upper M2 length (M2L), maximum cranial width (MCW), zygomatic width (ZW), least cranial width (LCW), cranial depth (CD), premaxilla depth (PD), mandible height (MH), mandible length (ML), lower M1 length (M1L), basicranial length (BCL). The cranioskeletal diagram was reproduced with author permission from Wayne, R. (Evolution 40, 243–261, 1986). (0.45 MB PDF) [file pbio.1000451.s001.pdf]

## Slide 1
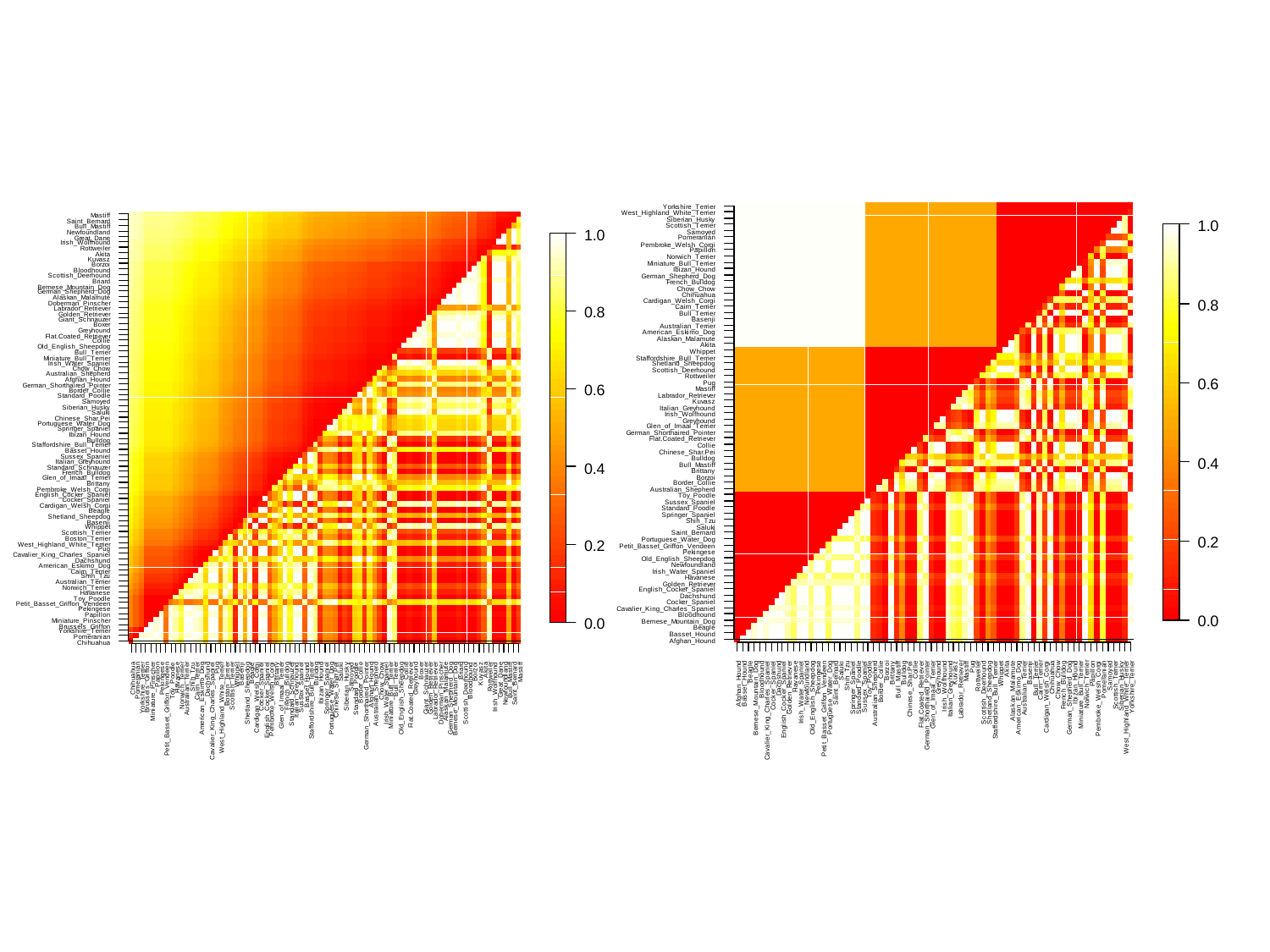

Supplement: Figure S2 — Correlation between the allele frequency of the most highly associated SNP (lower diagonal) and the phenotype for (A) log(body weight) and (B) ear floppiness (upper diagonal) across the 80 CanMap breeds. (0.24 MB PPT) [file pbio.1000451.s002.ppt]

Breed Weight

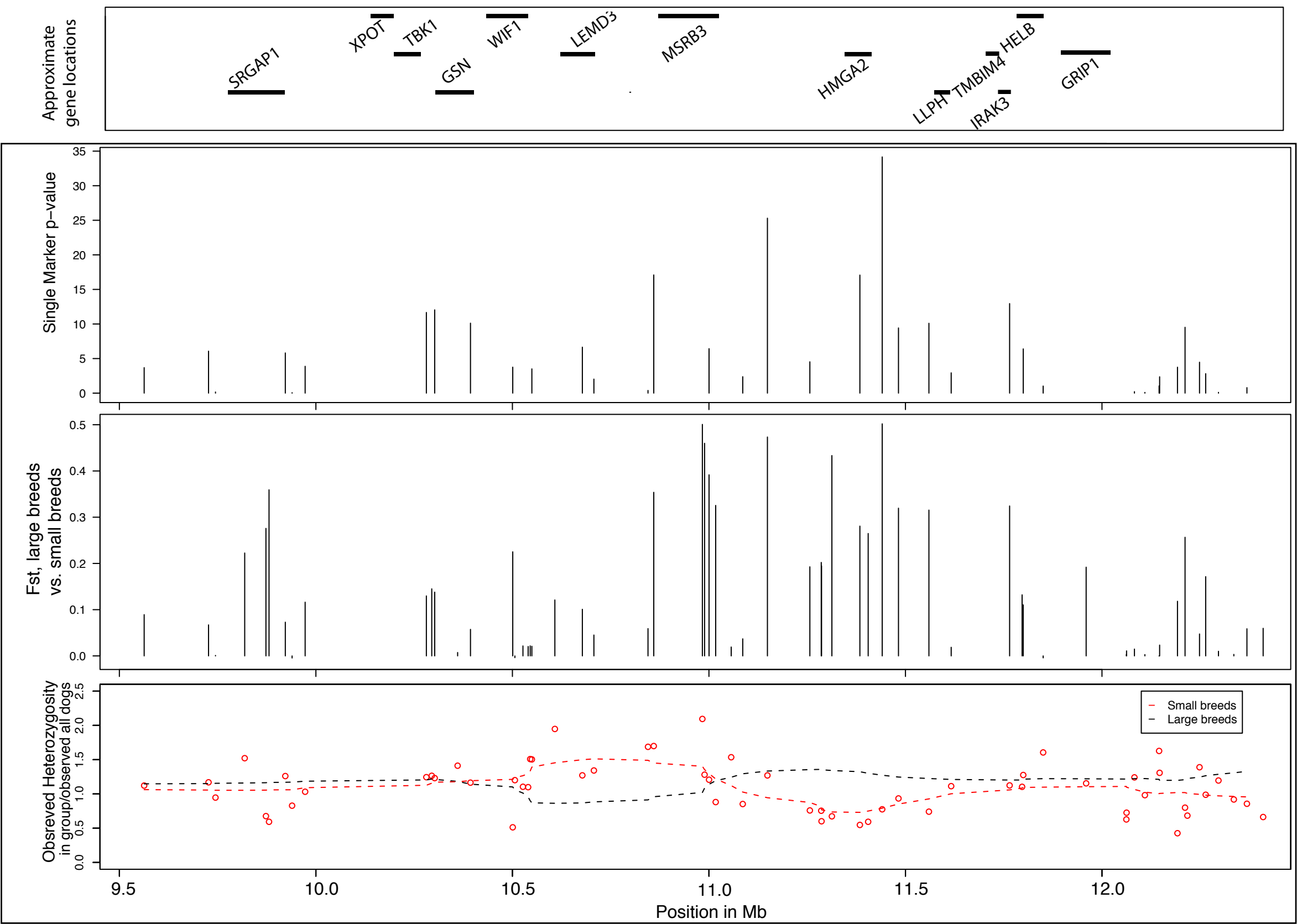

Ear-flop

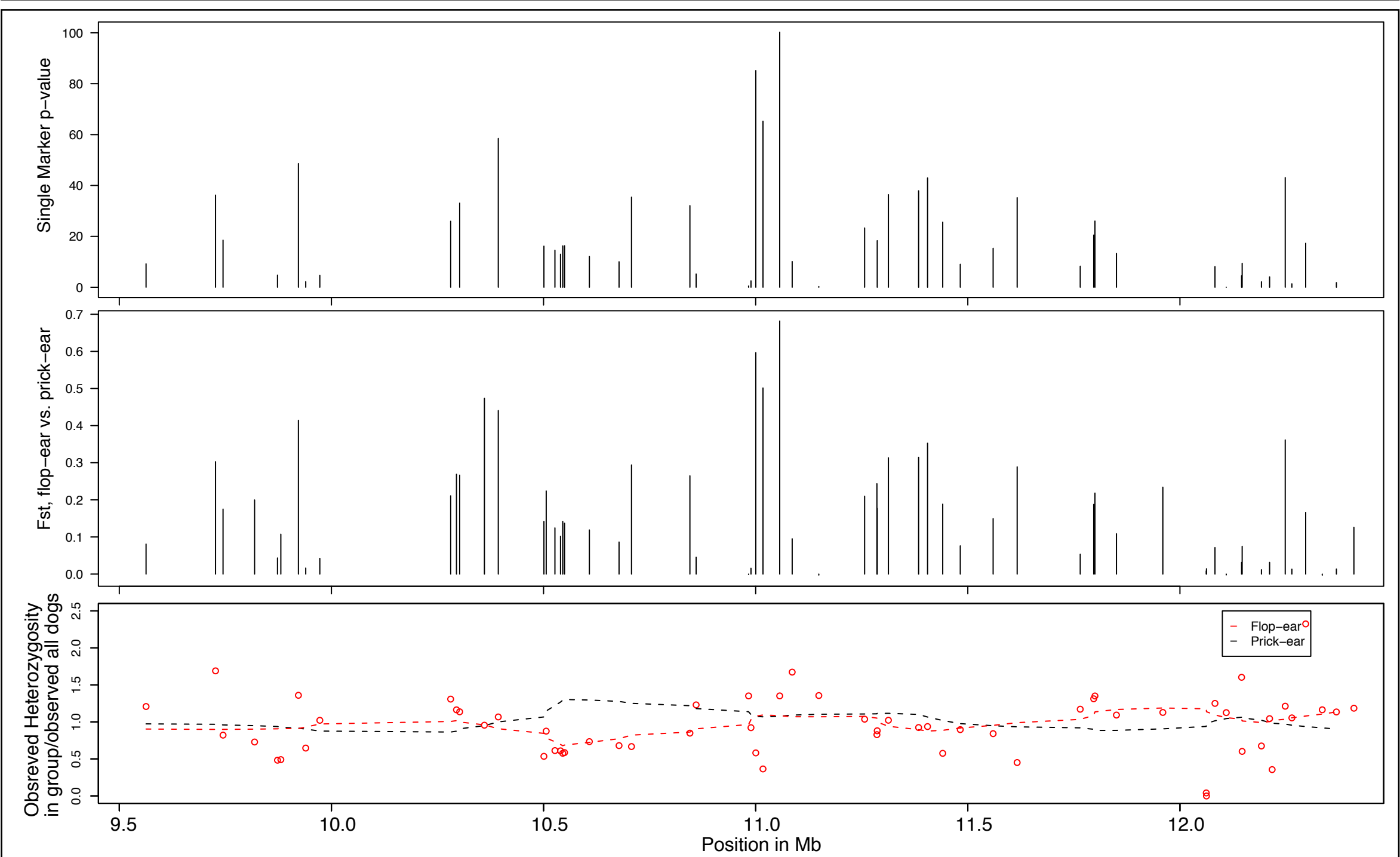

r-squared

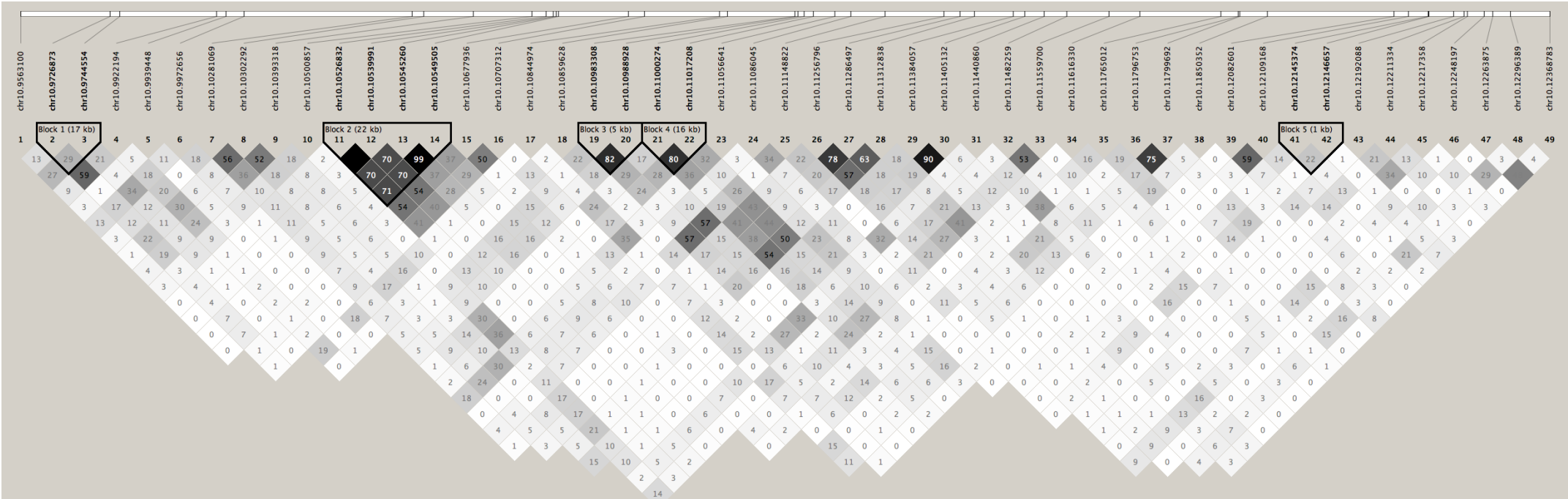

Supplement: Figure S3 — Fine-scale resolution of CFA10 region associated with both body size traits and ear floppiness. Single-marker analysis shows strongest association with body weight near HMGA2, while the strongest association with ear floppiness is near MSRB3. High FST between small- and large-breed dogs and reduced heterozygosity in small breed dogs extends several hundred kb away from HMGA2. The strongest ear flop association and FST signal between erect- and floppy-eared breeds are relatively localized within 100 kb region near MSRB3, although reduced heterozygosity in floppy-eared breeds extends for 500 kb. (0.49 MB PDF) [file pbio.1000451.s003.pdf]

## Slide 1
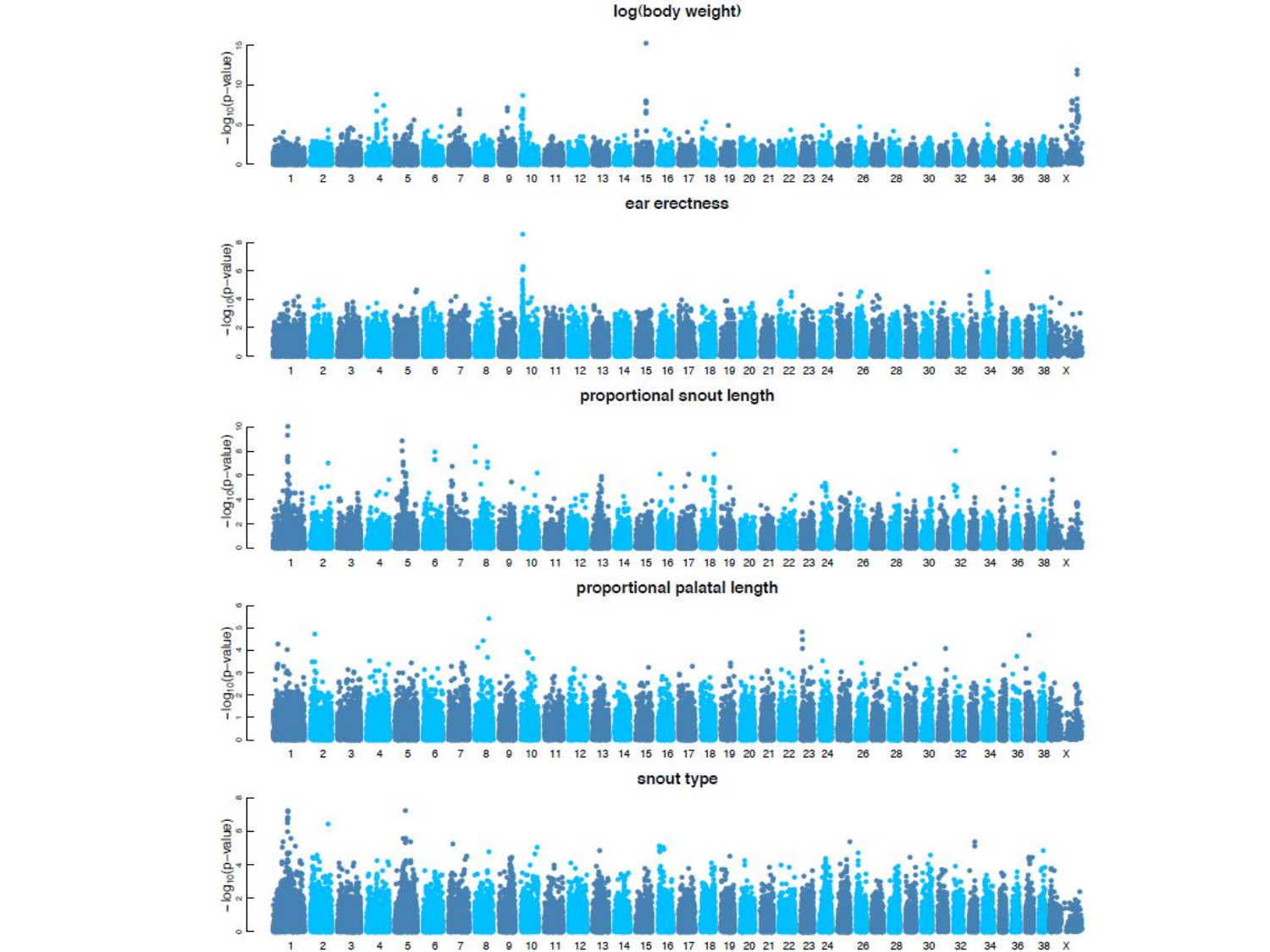

Supplement: Figure S4 — Genome-wide association scans using naïve tests without accounting for breed relatedness. Scans show (A) log(body weight), (B) ear erectness (floppy versus erect ears), (C) proportional snout length, (D) proportional palatal length, and (E) snout type (brachycephalic versus average). (0.30 MB PPT) [file pbio.1000451.s004.ppt]

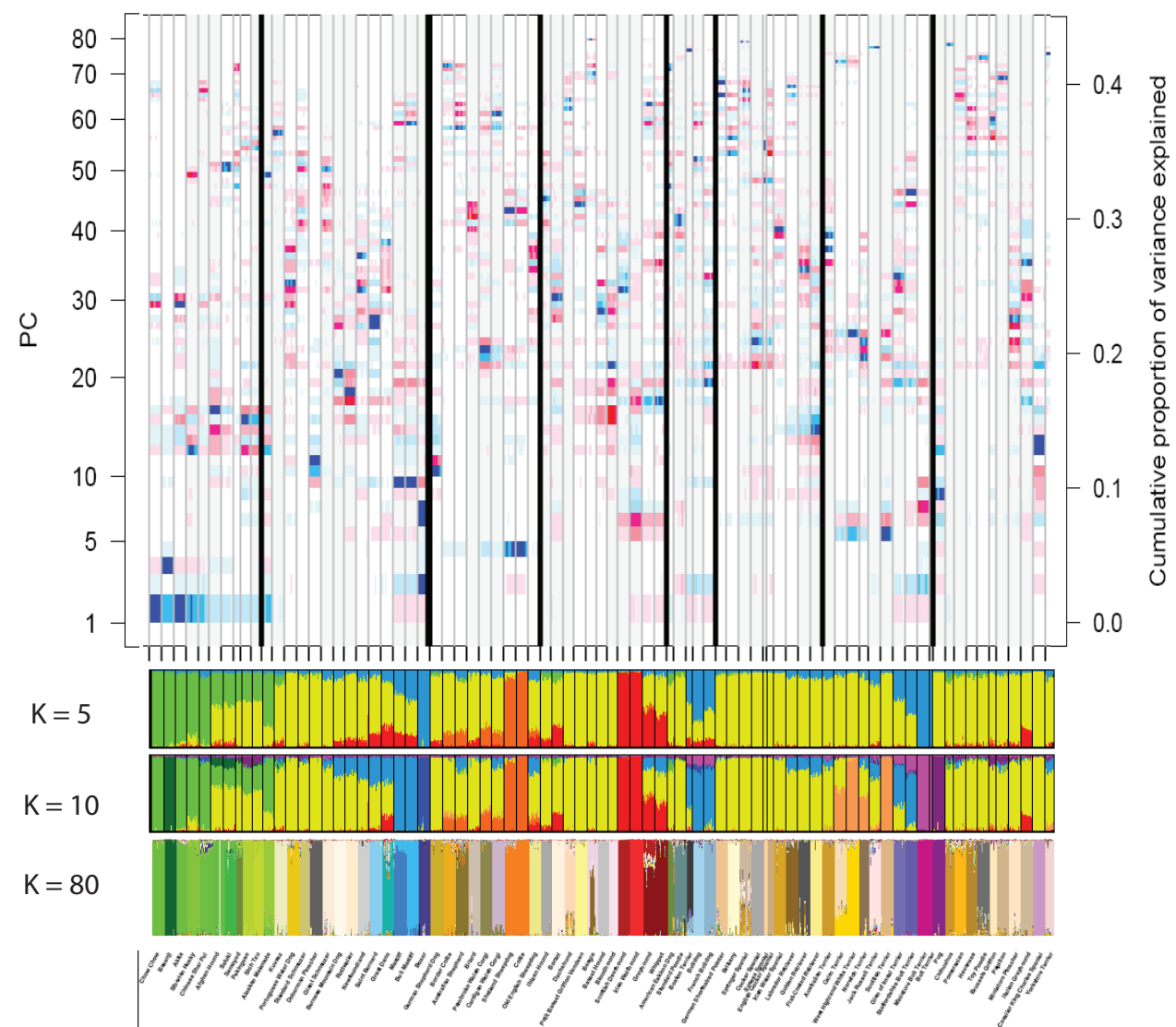

Supplement: Figure S5 — Population structure across CanMap breeds determined by PCA (top) and Structure (bottom). Each individual is a thin column and individuals are grouped by breed (black vertical lines separate breeds, with bold lines denoting separation between breed groups). Values for PC1 through PC80 are shown in descending order for each individual by color with blue indicating lower-than-average PC values and red indicating higher-than-average values. The height of each PC is proportional to the proportion of variance explained by each principal component (shown on right axis). Ordering of individuals along the x-axis (6–12 individuals per breed) is identical for both panels. (0.47 MB PDF) [file pbio.1000451.s005.pdf]
