# Supplementary material for: A Simple Genetic Architecture Underlies Morphological Variation in Dogs
Source: PLoS Biol. 2010 Aug 10;8(8):e1000451. doi: 10.1371/journal.pbio.1000451 (PMC2919785; doi:10.1371/journal.pbio.1000451)
Supplement: Table S1 — Proportion variance explained by models incorporating the top one to six SNPs for each trait. Blanks denote traits with too few significant SNPs to parameterize a full model. (0.10 MB DOC) [file pbio.1000451.s006.docx]

| **Trait** | **1-SNP** | **2-SNP** | **3-SNP** | **4-SNP** | **5-SNP** | **6-SNP** |
| --- | --- | --- | --- | --- | --- | --- |
| Abdominal Girth | 0.444 | 0.592 | 0.644 | 0.663 | 0.675 | 0.692 |
| Body Length | 0.537 | 0.614 | 0.670 | 0.703 | 0.726 | 0.742 |
| Chest Width | 0.310 | 0.507 | 0.570 | 0.606 | 0.620 | 0.631 |
| Eye Width | 0.389 | 0.487 | 0.551 | 0.583 | 0.599 | 0.604 |
| Forefoot Circumference | 0.360 | 0.537 | 0.603 | 0.641 | 0.664 | 0.674 |
| Forefoot Length | 0.439 | 0.571 | 0.633 | 0.675 | 0.702 | 0.725 |
| Head Length | 0.357 | 0.481 | 0.557 | 0.595 | 0.622 | 0.636 |
| Head Width | 0.336 | 0.377 | 0.411 | 0.430 | 0.447 | 0.454 |
| Height at Tail | 0.434 | 0.620 | 0.667 | 0.701 | 0.725 | 0.744 |
| Height at Withers | 0.444 | 0.624 | 0.674 | 0.713 | 0.741 | 0.763 |
| Hindfoot Circumference | 0.394 | 0.583 | 0.633 | 0.658 | 0.671 | 0.682 |
| Hindfoot Length | 0.450 | 0.608 | 0.656 | 0.697 | 0.727 | 0.746 |
| Lower Foreleg Length | 0.432 | 0.626 | 0.675 | 0.714 | 0.741 | 0.753 |
| Lower Hindleg Length | 0.413 | 0.600 | 0.651 | 0.695 | 0.721 | 0.735 |
| Neck Girth | 0.359 | 0.572 | 0.630 | 0.658 | 0.675 | 0.686 |
| Neck Length | 0.499 | 0.580 | 0.621 | 0.659 | 0.685 | 0.704 |
| Snout Length | 0.443 | 0.589 | 0.627 | 0.656 | 0.683 | 0.706 |
| Upper Foreleg Length | 0.462 | 0.613 | 0.658 | 0.693 | 0.721 | 0.738 |
| Upper Hindleg Length | 0.454 | 0.561 | 0.599 | 0.620 | 0.639 | 0.646 |
| Log(Body Weight) | 0.463 | 0.587 | 0.649 | 0.689 | 0.710 | 0.723 |
| Basicranial Length | 0.577 | 0.669 | 0.720 | 0.770 | 0.785 | 0.799 |
| Cranial Depth | 0.516 | 0.616 | 0.659 | 0.694 | 0.719 | 0.733 |
| Face Length | 0.655 | 0.711 | 0.740 | 0.757 | 0.774 | 0.783 |
| Least Cranial Width | 0.286 | 0.415 | 0.500 | 0.542 | 0.579 | 0.601 |
| Length Femur | 0.640 | 0.701 | 0.738 | 0.761 | 0.781 | 0.799 |
| Length Humerus | 0.656 | 0.716 | 0.750 | 0.773 | 0.794 | 0.805 |
| Length Metacarpal | 0.648 | 0.730 | 0.771 | 0.796 | 0.815 | 0.828 |
| Length Metatarsal | 0.655 | 0.698 | 0.731 | 0.757 | 0.773 | 0.786 |
| Length Olecranon | 0.655 | 0.696 | 0.743 | 0.765 | 0.785 | 0.800 |
| Length Radius | 0.644 | 0.712 | 0.749 | 0.771 | 0.787 | 0.803 |
| Length Scapula | 0.661 | 0.730 | 0.762 | 0.777 | 0.791 | 0.800 |
| Length Tibial Crest | 0.609 | 0.714 | 0.740 | 0.763 | 0.779 | 0.787 |
| Length Ulna | 0.660 | 0.725 | 0.761 | 0.785 | 0.805 | 0.822 |
| Lower M1 Length | 0.546 | 0.651 | 0.704 | 0.737 | 0.759 | 0.783 |
| Mandible Height | 0.557 | 0.625 | 0.660 | 0.690 | 0.707 | 0.725 |
| Mandible Length | 0.628 | 0.694 | 0.725 | 0.750 | 0.763 | 0.772 |
| Mandible Width | 0.437 | 0.647 | 0.694 | 0.713 | 0.724 | 0.733 |
| Maximum Cranial Width | 0.391 | 0.516 | 0.590 | 0.633 | 0.679 | 0.698 |
| Palatal Length | 0.671 | 0.715 | 0.736 | 0.758 | 0.771 | 0.781 |
| Palatal Width | 0.422 | 0.680 | 0.714 | 0.739 | 0.762 | 0.778 |
| Premaxilla Depth | 0.491 | 0.581 | 0.637 | 0.668 | 0.696 | 0.718 |
| Total Skull Length | 0.652 | 0.698 | 0.731 | 0.748 | 0.765 | 0.780 |
| Upper M1 Length | 0.698 | 0.726 | 0.751 | 0.774 | 0.789 | 0.798 |
| Upper M1 Width | 0.540 | 0.653 | 0.688 | 0.713 | 0.734 | 0.746 |
| Upper M2 Length | 0.605 | 0.670 | 0.692 | 0.709 | 0.724 | 0.737 |
| Upper M2 Width | 0.544 | 0.642 | 0.670 | 0.686 | 0.704 | 0.718 |
| Upper P3 Length | 0.406 | 0.492 | 0.553 | 0.572 | 0.589 | 0.600 |
| Upper P4 Length | 0.592 | 0.682 | 0.723 | 0.755 | 0.769 | 0.781 |
| Upper Tooth Row Length | 0.630 | 0.699 | 0.738 | 0.762 | 0.782 | 0.795 |
| Width Femur | 0.595 | 0.651 | 0.696 | 0.726 | 0.748 | 0.765 |
| Width Humerus | 0.471 | 0.581 | 0.644 | 0.676 | 0.702 | 0.715 |
| Width Infraspinous Fossa | 0.655 | 0.710 | 0.737 | 0.757 | 0.774 | 0.786 |
| Width Metacarpal | 0.528 | 0.624 | 0.673 | 0.691 | 0.710 | 0.726 |
| Width Metatarsal | 0.543 | 0.611 | 0.645 | 0.684 | 0.714 | 0.741 |
| Width Scapula | 0.632 | 0.680 | 0.710 | 0.738 | 0.756 | 0.769 |
| Zygomatic Width | 0.434 | 0.677 | 0.718 | 0.751 | 0.765 | 0.775 |
| Abdominal Girth, allometric | 0.090 |  |  |  |  |  |
| Body Length, allometric | 0.150 | 0.238 | 0.298 | 0.347 | 0.392 | 0.411 |
| Chest Width, allometric | 0.137 | 0.180 |  |  |  |  |
| Eye Width, allometric | 0.181 | 0.307 | 0.384 | 0.442 | 0.485 | 0.519 |
| Forefoot Circumference, allometric | 0.148 | 0.235 | 0.297 | 0.346 | 0.380 | 0.416 |
| Forefoot Length, allometric | 0.164 | 0.259 | 0.340 | 0.390 | 0.430 | 0.454 |
| Head Length, allometric | 0.125 | 0.213 | 0.268 | 0.307 | 0.322 |  |
| Head Width, allometric | 0.160 | 0.265 | 0.345 | 0.412 | 0.459 | 0.491 |
| Height at Tail, allometric | 0.218 | 0.314 | 0.387 | 0.441 | 0.480 | 0.507 |
| Height at Withers, allometric | 0.210 | 0.306 | 0.387 | 0.434 | 0.477 | 0.522 |
| Hindfoot Circumference, allometric | 0.122 | 0.205 | 0.264 | 0.320 | 0.365 | 0.411 |
| Hindfoot Length, allometric | 0.191 | 0.284 | 0.374 | 0.433 | 0.472 | 0.504 |
| Lower Foreleg Length, allometric | 0.248 | 0.354 | 0.436 | 0.482 | 0.527 | 0.562 |
| Lower Hindleg Length, allometric | 0.198 | 0.307 | 0.378 | 0.426 | 0.468 | 0.511 |
| Neck Girth, allometric | 0.169 | 0.261 | 0.338 | 0.392 | 0.436 | 0.479 |
| Neck Length, allometric | 0.192 | 0.309 | 0.370 | 0.415 | 0.455 | 0.484 |
| Snout Length, allometric | 0.333 | 0.490 | 0.543 | 0.569 | 0.595 | 0.611 |
| Upper Foreleg Length, allometric | 0.167 | 0.268 | 0.347 | 0.390 | 0.435 | 0.473 |
| Upper Hindleg Length, allometric | 0.142 | 0.267 | 0.330 | 0.376 | 0.413 | 0.443 |
| Length Femur, allometric | 0.213 | 0.334 | 0.417 | 0.459 | 0.496 | 0.519 |
| Length Humerus, allometric | 0.224 | 0.340 | 0.429 | 0.490 | 0.512 | 0.531 |
| Length Metacarpal, allometric | 0.272 | 0.390 | 0.481 | 0.536 | 0.571 | 0.588 |
| Length Metatarsal, allometric | 0.232 | 0.354 | 0.429 | 0.490 | 0.519 | 0.540 |
| Length Olecranon, allometric | 0.144 | 0.251 | 0.318 | 0.364 | 0.399 | 0.428 |
| Length Radius, allometric | 0.229 | 0.356 | 0.433 | 0.499 | 0.554 | 0.584 |
| Length Scapula, allometric | 0.278 | 0.462 | 0.574 | 0.616 | 0.646 | 0.668 |
| Length Tibial Crest, allometric | 0.205 | 0.325 | 0.396 | 0.426 | 0.448 |  |
| Length Ulna, allometric | 0.226 | 0.343 | 0.418 | 0.474 | 0.499 | 0.521 |
| Total Skull Length, allometric | 0.207 | 0.295 | 0.359 | 0.403 | 0.447 | 0.465 |
| Width Femur, allometric | 0.212 | 0.304 | 0.363 | 0.400 | 0.435 | 0.455 |
| Width Humerus, allometric | 0.231 | 0.387 | 0.471 | 0.517 | 0.549 | 0.570 |
| Width Infraspinous Fossa, allometric | 0.248 | 0.341 | 0.405 | 0.448 | 0.488 | 0.522 |
| Width Metacarpal, allometric | 0.160 | 0.251 | 0.314 | 0.361 | 0.391 |  |
| Width Metatarsal, allometric | 0.129 | 0.220 | 0.285 |  |  |  |
| Width Scapula, allometric | 0.262 | 0.393 | 0.482 | 0.562 | 0.623 | 0.651 |
| Basicranial Length, allometric | 0.239 | 0.374 | 0.408 | 0.434 | 0.458 | 0.466 |
| Cranial Depth, allometric | 0.306 | 0.413 | 0.478 | 0.512 | 0.537 | 0.556 |
| Face Length, allometric | 0.019 |  |  |  |  |  |
| Least Cranial Width, allometric | 0.268 | 0.416 | 0.499 | 0.563 | 0.603 | 0.643 |
| Lower M1 Length, allometric | 0.293 | 0.421 | 0.468 | 0.500 | 0.525 | 0.535 |
| Mandible Height, allometric | 0.224 | 0.322 | 0.378 | 0.417 | 0.438 | 0.448 |
| Mandible Length, allometric | 0.289 | 0.326 | 0.340 |  |  |  |
| Mandible Width, allometric | 0.316 | 0.398 | 0.474 | 0.523 | 0.549 | 0.561 |
| Maximum Cranial Width, allometric | 0.233 | 0.358 | 0.418 | 0.467 | 0.501 | 0.523 |
| Palatal Length, allometric | 0.141 | 0.206 | 0.241 | 0.257 |  |  |
| Palatal Width, allometric | 0.413 | 0.527 | 0.594 | 0.634 | 0.672 | 0.690 |
| Premaxilla Depth, allometric | 0.386 | 0.439 | 0.470 | 0.495 | 0.513 | 0.531 |
| Upper M1 Length, allometric | 0.090 | 0.143 |  |  |  |  |
| Upper M1 Width, allometric | 0.273 | 0.398 | 0.461 | 0.490 | 0.503 | 0.512 |
| Upper M2 Length, allometric | 0.115 | 0.154 |  |  |  |  |
| Upper M2 Width, allometric | 0.178 | 0.269 | 0.361 | 0.395 | 0.425 | 0.443 |
| Upper P3 Length, allometric | 0.118 | 0.191 |  |  |  |  |
| Upper P4 Length, allometric | 0.256 | 0.349 | 0.414 | 0.445 | 0.459 |  |
| Upper Tooth Row Length, allometric | 0.207 | 0.303 | 0.343 | 0.376 | 0.403 |  |
| Zygomatic Width, allometric | 0.361 | 0.531 | 0.576 | 0.606 | 0.627 | 0.644 |
