# Supplementary material for: A Simple Genetic Architecture Underlies Morphological Variation in Dogs
Source: PLoS Biol. 2010 Aug 10;8(8):e1000451. doi: 10.1371/journal.pbio.1000451 (PMC2919785; doi:10.1371/journal.pbio.1000451)
Supplement: Table S2 — Comparison of BRLMM-P and MAGIC genotype calling algorithms using common Affymetrix .cel files and QC filters. Note that the 1,400 arrays used for the analyses in this study are a subset of the arrays used to conduct this head-to-head comparison, so total SNP counts differ somewhat between the datasets. (0.05 MB DOC) [file pbio.1000451.s007.docx]

|  | BRLMM-P | MAGIC |
| --- | --- | --- |
| Putative SNPs on Array | 127,132 | 127,132 |
| Called by Algorithm | 127,132 | 87,317 |
| Excluded due to high missingness | 65,160 | 25,165 |
| Excluded due to extreme HWE deviations | 659 | 1,551 |
| Removed due to high discordance | 20,655 | 1,186 |
|  |  |  |
| Final SNP Count | **40,658** | **57,938** |
| Call Rate | **97.94%** | **95.83%** |
| Concordance | **99.57%** | **99.89%** |
