# Supplementary material for: A Simple Genetic Architecture Underlies Morphological Variation in Dogs
Source: PLoS Biol. 2010 Aug 10;8(8):e1000451. doi: 10.1371/journal.pbio.1000451 (PMC2919785; doi:10.1371/journal.pbio.1000451)
Supplement: Table S3 — List of SNPs that were sequenced to validate the MAGIC genotyping algorithm. Red SNPs indicate discordant homozygous calls between MAGIC and BRLMM, which are indicative of the presence of “null alleles” (individuals lacking specific binding to either probe, usually because of a variant at the probe binding site). (0.06 MB DOC) [file pbio.1000451.s008.docx]

| Chromosome | Position | Concordant  calls | Discordant  calls | Homo-homo^1^ | Homo-hetero^2^ | Other polymorphism |
| --- | --- | --- | --- | --- | --- | --- |
| 1 | 113380161 | 95 | 603 | 0 | 603 |  |
| 1 | 121518445 | 277 | 464 | 0 | 464 |  |
| 2 | 77541290 | 87 | 611 | 0 | 611 |  |
| 5 | 56886123 | 399 | 370 | 22 | 348 | indel |
| 8 | 24865845 | 452 | 120 | 50 | 70 | SNP |
| 14 | 45369804 | 8 | 513 | 3 | 510 | SNP |
| 17 | 13910226 | 88 | 643 | 0 | 643 |  |
| 22 | 30233920 | 92 | 590 | 0 | 590 |  |
| 24 | 20837991 | 127 | 539 | 11 | 528 | indel |
| 27 | 28775379 | 705 | 64 | 13 | 51 | SNP |
| 35 | 4345196 | 767 | 28 | 13 | 15 | SNP |
| 36 | 29262269 | 725 | 35 | 13 | 22 | SNP |
