# Supplementary material for: A Simple Genetic Architecture Underlies Morphological Variation in Dogs
Source: PLoS Biol. 2010 Aug 10;8(8):e1000451. doi: 10.1371/journal.pbio.1000451 (PMC2919785; doi:10.1371/journal.pbio.1000451)
Supplement: Text S1 — Algorithmic details and validation of MAGIC genotype calling program. (0.12 MB DOC) [file pbio.1000451.s009.docx]

In the first stage of the MAGIC algorithm, we perform quantile normalization of the data, as is common for current algorithms. This process is performed for each chip independently, and hence is parallelizable. We found that additional, more sophisticated, normalizations do not significantly aid the genotype calling process.

The main stage of the algorithm proceeds by performing a Principal Component Analysis (PCA) of the normalized intensity data from all individuals on a SNP-by-SNP basis. This method neatly summarizes the raw data so that genotypes can be called using standard clustering techniques. We use an Expectation Maximization algorithm to fit a t-distribution mixture model to cluster the data into genotype calls. We start by fitting three t-distributions using the first two principal components since the majority of SNPs can be clustered cleanly using the first two principal components. However, it is possible to cluster using additional components, which can help in cases where the algorithm gets stuck in suboptimal solutions. This stage of the algorithm is computationally intensive, but each SNP can be processed independently, and hence is parallelizable. The clusters can then be assigned to genotypes based on the average binding strength to each probe type for individuals in the cluster.

MAGIC outperforms BRLMM-P by allowing more SNPs to be genotyped on the array, with higher concordance between duplicate assays when using identical QC thresholds (in particular, requiring SNPs to have call rates >70%, heterozygosity rates between 20% and 90% that expected under HWE, and concordance rates of samples run in duplicate of >97%, see Table S2). However, concordance is not proof of genotyping accuracy (a genotyping algorithm can give an incorrect genotyping call consistently across replicates), so we investigated twelve SNPs showing call discrepancies between MAGIC and BRLMM-P (Table S3). Out of 36,053 SNPs that were called by the two algorithms, 13061 had at least one discordant call on the 809 samples analyzed. We designed primers using Primer3 [48] and amplified each amplicon by PCR using standard protocols and a 40 cycle, touch-down thermocycler program with the annealing temperature reduced by 1/2 a degree for the first 20 cycles, then held steady at the lowest temperature for the last 20 cycles. PCR products were sequenced using BigDye terminator sequencing kits and standard protocols (Applied Biosystems, Santa Clara, CA). For each SNP, we sequenced eight dogs, two dogs with concordant genotypes and six with discordant genotypes. We then compared the genotypes of BRLMM-P and MAGIC with the genotypes of the sequences.

For five SNPs, all discordant genotypes were changing from a homozygous status to a heterozygous status. In those five SNPs, the sequences showed that BRLMM-P miscalled the genotypes and that MAGIC called the right genotypes. For the other seven SNPs, some discordant genotypes were homozygous for one allele in one caller and homozygous for the other allele in the other. In those cases, neither BRLMM-P nor MAGIC was performing better to call the right genotypes. The sequencing revealed that for those SNPs, at least one other polymorphism (SNP or indel) was in very close vicinity of the tested SNP. In the CanMap project, 62 SNPs had discrepant calls of this type, and were then removed from the dataset.

In addition, we required SNPs to have call rates >70%, heterozygosity rates between 20% and 90% that expected under HWE, and concordance rates of samples run in duplicate of >97% (Table S2).

As a final quality control step, we applied the hidden Markov model described in [16] to detect genomic regions of autozygosity within each of the 1400 CanMap individuals. Since mean autozygosity was above 20% in the dataset, we expected nearly 300 individuals to be within an autozygous segment at any SNP on the array. All of these ~300 individuals should have homozygous genotype calls for that SNP, although in practice some heterozygous calls can be expected owing to gene conversion or imperfect inference of the autozygous segments. SNPs with poor genotyping quality, specifically SNPs with a spurious excess of heterozygous calls, will exhibit relatively high rates of heterozyosity even within inferred segments of autozygosity. We excluded 451 SNPs with elevated heterozygosity within autozygous segments (here defined as >10%). Visual inspection of the cluster plots suggested many of these SNPs occurred within segmental duplications or copy number variable regions, or contained a substantial fraction of null alleles mistakenly called as heterozygous.
